# Rapid Polymerase Chain Reaction–Based Test for the Detection of Female Urogenital Chlamydial Infections

**DOI:** 10.1155/S1064744994000050

**Published:** 1994

**Authors:** Harold C. Wiesenfeld, Michael Uhrin, Bruce W. Dixon, Richard L. Sweet

**Affiliations:** 1 Department of Obstetrics, Gynecology, and Reproductive Sciences Magee-Womens Hospital University of Pittsburgh School of Medicine 300 Halket Street Pittsburgh PA 15213 USA; 2 Department of Medicine University of Pittsburgh School of Medicine Pittsburgh PA USA

## Abstract

*Objective:* The purpose of this study was to evaluate the Amplicor *Chlamydia trachomatis* Test (Roche Molecular Systems, Branchburg, NJ), a polymerase chain reaction (PCR)-based technique, as a screening test for the detection of female urogenital *C. trachomatis* infections, comparing it to an enzyme immunoassay method.

*Methods:* Endocervical specimens for PCR and Chlamydiazyme (Abbott Laboratories, North Chicago, IL) analysis were obtained from 328 unselected patients at the outpatient Sexually Transmitted Diseases Clinic at the Allegheny County Health Department, Pittsburgh, PA. In addition, urethral swabs for PCR analysis were obtained from 256 of these patients.

*Results:* By PCR analysis, the prevalence of urogenital chlamydial infections was 15.6% and that of cervical chlamydial infections was 10.7%. The sensitivity of PCR in the detection of endocervical chlamydial infections was 89.7% and the specificity was 100%. The positive and negative predictive values of PCR were 100% and 99%, respectively. The sensitivity of Chlamydiazyme in the detection of cervical infections was 61.5% and the specificity was 99.7%, with a positive predictive value of 96.0%. Among all patients with urogenital chlamydial infections, concomitant infections in the urethra and cervix occurred in 52.5%, whereas the urthra or cervix was solely infected in 35.0% and 12.5%, respectively.

*Conclusions:* This PCR-based technique is a rapid screening tool for the diagnosis of urogenital chlamydial infections and is more sensitive than Chlamydiazyme for endocervical infections in a sexually transmitted disease clinic population.


Infectious Diseases in Obstetrics and Gynecology 1:182–187 (1994)
© 1994 Wiley-Liss, Inc.

### Rapid Polymerase Chain Reaction–Based Test for the Detection of Female Urogenital Chlamydial Infections

**Harold C. Wiesenfeld, Michael Uhrin, Bruce W. Dixon, and Richard L. Sweet**

*Department of Obstetrics, Gynecology, and Reproductive Sciences, Magee-Womens Hospital (H.C.W., M.U., R.L.S.), and Department of Medicine (B.W.D.), University of Pittsburgh School of Medicine, Pittsburgh, PA*

### Abstract

***Objective:*** The purpose of this study was to evaluate the Amplicor *Chlamydia trachomatis* Test (Roche Molecular Systems, Branchburg, NJ), a polymerase chain reaction (PCR)-based technique, as a screening test for the detection of female urogenital *C. trachomatis* infections, comparing it to an enzyme immunoassay method.

***Methods:*** Endocervical specimens for PCR and Chlamydiazyme (Abbott Laboratories, North Chicago, IL) analysis were obtained from 328 unselected patients at the outpatient Sexually Transmitted Diseases Clinic at the Allegheny County Health Department, Pittsburgh, PA. In addition, urethral swabs for PCR analysis were obtained from 256 of these patients.

***Results:*** By PCR analysis, the prevalence of urogenital chlamydial infections was 15.6% and that of cervical chlamydial infections was 10.7%. The sensitivity of PCR in the detection of endocervical chlamydial infections was 89.7% and the specificity was 100%. The positive and negative predictive values of PCR were 100% and 99%, respectively. The sensitivity of Chlamydiazyme in the detection of cervical infections was 61.5% and the specificity was 99.7%, with a positive predictive value of 96.0%. Among all patients with urogenital chlamydial infections, concomitant infections in the urethra and cervix occurred in 52.5%, whereas the urethra or cervix was solely infected in 35.0% and 12.5%, respectively.

***Conclusions:*** This PCR-based technique is a rapid screening tool for the diagnosis of urogenital chlamydial infections and is more sensitive than Chlamydiazyme for endocervical infections in a sexually transmitted disease clinic population. © 1994 Wiley-Liss, Inc.

Key words

Cervicitis, urethritis, PCR-based test, chlamydial infections

*Chlamydia trachomatis*, an obligatory intracellular bacterium, is one of the most common sexually transmitted organisms in developed countries. The prevalence of chlamydial infections varies, depending on the population examined, from an estimated 4–5% of all sexually active women in the United States to as many as 33% of patients attending sexually transmitted disease (STD) clinics.[1] In women, *C. trachomatis* is a major cause of urethritis, cervicitis, and pelvic inflammatory disease. Long-term sequelae of upper genital tract infections include infertility, ectopic pregnancy, and chronic pelvic pain. Recent chlamydial infections may be associated with premature rupture of membranes, preterm labor, and late postpartum endomyometritis.[2–4] In men, *C. trachomatis* causes over 40% of cases of non-gonococcal urethritis[5] and is an important cause of epididymitis, prostatitis, and proctitis. Vertical transmission of *C. trachomatis* can lead to inclusion conjunctivitis in the newborn

Address correspondence/reprint requests to Dr. Harold C. Wiesenfeld, Department of Obstetrics, Gynecology, and Reproductive Sciences, Magee-Womens Hospital, 300 Halket Street, Pittsburgh, PA 15213.

Received November 17, 1993
Accepted January 8, 1994

Basic Science Article and pneumonia in infants during the first months of life. In addition to the clinical manifestations mentioned, there is a high rate of asymptomatic infection in both males and females; up to 70% of infected females are asymptomatic.[6]

There are a variety of methods available for the detection of *C. trachomatis*. While the currently accepted gold standard diagnostic test is tissue culture isolation of the organism, its sensitivity is estimated to be only 80%.[7] Moreover, it requires up to 72 h for incubation and is costly. Antigen detection methods, based on fluorescent monoclonal antibody (MicroTrak, Syva, Palo Alto, CA) or enzyme-linked immunoassay (Chlamydiazyme, Abbott Laboratories, North Chicago, IL) methods are widely used in detecting chlamydial infections. These tests are less expensive and can be performed more rapidly than cell culture; however, they are less sensitive and specific.[8]

The polymerase chain reaction (PCR) detects small quantities of deoxyribonucleic acid (DNA) by using a DNA polymerase to amplify a target DNA sequence. The PCR technique has been developed to detect viral and bacterial infections. PCR detection of *C. trachomatis* infections has been evaluated in several small studies.[9–11] This technique, however, is time consuming and labor intensive. A rapid PCR assay for the detection of *C. trachomatis* has been developed recently (Amplicor *C. trachomatis* Test, Roche Molecular Systems, Branchburg, NJ). The Amplicor test has been demonstrated to be more sensitive than culture for the detection of endocervical infections in a high-prevalence obstetric population and more sensitive than Chlamydiazyme in a low-prevalence population.[12] This technique offers both excellent sensitivity and a total assay time of 4.5 h, which are important characteristics for a diagnostic or screening tool. In this study, we evaluated the Amplicor test on lower genital tract specimens from female patients attending an STD clinic and compared endocervical specimens with Chlamydiazyme, the primary diagnostic test for *C. trachomatis* used at our institution.

### SUBJECTS AND METHODS

Our study population was comprised of patients attending the outpatient Sexually Transmitted Diseases Clinic at the Allegheny County Health Department, Pittsburgh, PA. Specimens were obtained from both symptomatic and asymptomatic women from September 1, 1992, to October 30, 1992. Patients were included in the study if one of the investigators (H.C.W.) or certain nurse clinicians were present to collect the specimens. Cervical swabs were obtained randomly from 328 unselected female patients attending the clinic, and urethral swabs were obtained concomitantly from 256 of these patients.

Urethral specimens were obtained by passing a Dacron™-tipped swab into the distal urethra. A non-lubricated speculum was then placed into the vagina, excess cervical mucus was removed, and then endocervical samples were obtained for culture of *Neisseria gonorrhoeae*, Chlamydiazyme, and PCR (Amplicor *C. trachomatis* Test), in that order. Cervical swabs for Chlamydiazyme were obtained prior to those for the Amplicor test in order to obtain the greatest bacterial inoculum to facilitate antigen detection. The Chlamydiazyme specimens were processed as per the manufacturer's recommendation. The PCR Dacron™ swabs were placed in 1 ml of sodium dodecyl sulfate-based specimen transport media and agitated for 15 sec to displace the clinical sample into the media. The PCR specimens were sent at room temperature to a single research laboratory at Magee-Womens Hospital, Pittsburgh, PA, and these specimens were maintained at 4°C prior to processing.

PCR amplification was performed on clinical specimens, negative controls, and positive plasmid DNA controls according to a procedure described elsewhere.[12] Briefly, 50 μl of each diluted specimen was placed in a PCR reaction tube, to which was added a 50 μl aliquot of master mix containing Taq polymerase, uracil-N-glycosylase, Tris buffer, KCl, glycerol, deoxynucleotide triphosphates, and biotinylated oligonucleotide primers specific for the cryptic plasmid of *C. trachomatis*. A 2-temperature, 30-cycle amplification scheme was then conducted in a Perkin Elmer (Norwalk, CT) 9600 thermal cycler.

Sodium hydroxide-based denaturation solution was added to the amplified specimens whereupon 25 μl of each specimen was removed and placed into a 96-well microtiter detection plate containing a hybridization buffer. This microtiter plate was coated with a complementary probe for the amplified chlamydia cryptic plasmid. The amplified DNA was then permitted to hybridize, and the wells were then washed in order to remove unhy-

**TABLE 1. Comparison of Chlamydiazyme to PCR for the detection of endocervical *C. trachomatis***

| Chlamydiazyme results | PCR results | | Total |
|---|---|---|---|
| | Positive | Negative | |
| Reactive | 20 | 5 | 25 (7.6%) |
| Non-reactive | 15 | 288 | 303 |
| Total | 35 (10.7%) | 293 | 328 |

**TABLE 2. Performance of PCR in the detection of endocervical *C. trachomatis* (after resolution of discrepant results)**

| PCR results | Resolved specimens | | Total |
|---|---|---|---|
| | Positive | Negative | |
| Positive | 35 | 0 | 35 |
| Negative | 4 | 289 | 293 |
| Total | 39 | 289 | 328 |

**TABLE 3. Performance of Chlamydiazyme in the detection of endocervical *C. trachomatis* (after resolution of discrepant results)**

| Chlamydiazyme results | Resolved specimens | | Total |
|---|---|---|---|
| | Positive | Negative | |
| Positive | 24 | 1 | 25 |
| Negative | 15 | 288 | 303 |
| Total | 39 | 289 | 328 |

bridized excess amplification reagents. Avidin-horseradish peroxidase conjugate was added to each well, followed by a wash to remove unbound conjugate. Horseradish peroxidase substrate, containing hydrogen peroxide and tetramethylbenzidine, was then added for 10 min. The optical densities were read at 450 nm in a Biotek ELISA plate reader (Winooski, VT). Following preestablished criteria,[12] we graded absorbencies ≥0,25 as positive. Specimens with initial absorbencies between 0,20 and 0,60 were deemed equivocal and retested, with the second amplification value used as the final result.

Discrepant results were analyzed in the following manner. Specimens that were positive by PCR and negative by Chlamydiazyme were reamplified using primers directed against the major outer membrane protein (MOMP) gene of *C. trachomatis*, as described elsewhere.[12] A negative result after reamplification indicated contamination by cryptic plasmid amplicons, whereas a positive result indicated a false negative Chlamydiazyme result. Specimens positive by Chlamydiazyme and negative by PCR were reamplified after phenol-chloroform, ethanol-precipitated extraction. A negative result indicated sampling error, infection with a plasmidless strain of *C. trachomatis*, or a Chlamydiazyme false positive. A positive result after extraction signified the presence of PCR inhibitors.

### RESULTS

The results of Chlamydiazyme and Amplicor *C. trachomatis* Tests on cervical specimens are compared in Table 1. Twenty-five patients (7.6%) tested positive for *C. trachomatis* by Chlamydiazyme, and 35 patients (10.7%) were positive by PCR analysis. Compared to PCR, the sensitivity of Chlamydiazyme was only 57.1% and the specificity was 98.3%. The positive predictive value (PPV) of Chlamydiazyme was 80.0% and the negative predictive value (NPV) was 95.0%.

Discrepant results were noted in 20 specimens. Fifteen patients were positive by PCR and negative by Chlamydiazyme. All 15 specimens were confirmed as positive after amplification with primers for the MOMP gene. Four of the 5 specimens positive by Chlamydiazyme and negative by PCR were resolved as positive. One specimen remained negative after phenol-chloroform extraction.

The results of PCR and Chlamydiazyme after resolution of discrepant results are shown in Tables 2 and 3. The sensitivity of PCR in detecting endocervical *C. trachomatis* infections was 89.7% and the specificity was 100%. The PPV and NPV of PCR were 100% and 98.6%, respectively. Chlamydiazyme demonstrated a sensitivity of 61.5% and a specificity of 99.7%. The PPV and NPV were 96.0% and 95.0%, respectively. The difference in sensitivities of PCR and Chlamydiazyme was significant ($P < 0.01$, $\chi^2$ analysis).

Table 4 displays the results of the Amplicor test from patients with both urethral and cervical specimens. These paired samples were separately run using the PCR technique. *C. trachomatis* was identified in the cervix in 26 women (10.2%) and urethral chlamydia infections were detected in 35 patients (13.7%). Forty patients (15.6%) were

**TABLE 4. Comparison of the detection of cervical and urethral *C. trachomatis* by PCR**

| Urethra | Cervix Positive | Cervix Negative | Total |
|---|---|---|---|
| Positive | 21 | 14 | 35 (13.7%) |
| Negative | 5 | 216 | 221 |
| Total | 26 (10.2%) | 230 | 256 |

positive for chlamydia in either the cervix or urethra. Twenty-one of 26 patients (80.8%) with cervical chlamydial infections had concomitant urethral chlamydia infections. Likewise, of 35 patients with positive urethral PCR samples, 21 (60.0%) were positive for chlamydia in the cervix. Of all patients with urogenital chlamydial infections, *C. trachomatis* was identified in both sites in 21 (52.5%), while 14 (35.0%) were positive only in the urethra and only 5 (12.5%) were positive solely in the cervix.

Forty-two women were positive for *N. gonorrhoeae* in the endocervix. Cervical chlamydial infections were detected in 15 (35.7%) of these patients using PCR, whereas 10 patients (23.8%) were positive by Chlamydiazyme. Among the 35 patients positive for *C. trachomatis* by PCR, cervical coinfection with *N. gonorrhoeae* occurred in 15 (42.9%).

### DISCUSSION

Our study demonstrates the superior sensitivity of PCR, using the Amplicor *C. trachomatis* Test, when compared to enzyme immunoassay (Chlamydiazyme) in the detection of cervical chlamydial infections in women attending an STD clinic. The sensitivity of PCR in our study was 89.7%, whereas the sensitivity of Chlamydiazyme was 61.5% ($P < 0.01$). In our high-risk population, the PCR technique detected 15 additional patients who were negative by enzyme immunoassay. Over 35% of those patients infected were not identified by Chlamydiazyme. Chlamydiazyme detected 5 patients not identified by PCR. Four of these specimens were resolved as positive, whereas 1 remained negative after discrepant analysis. The 4 false negative PCR specimens were among the first 15% of samples analyzed, and we speculate that this may represent inexperience in the early phases of the learning curve.

Using PCR, the prevalences of chlamydial cervicitis and urethritis in our population are 10.7% and 13.7%, respectively, and the prevalence of urogenital chlamydial infections is 15.6%. Our data indicate that there is a high rate of concomitant urethral and cervical chlamydial infections and confirm other screening studies in STD clinics that suggest that 50% of women with urogenital chlamydial infections are infected in both the cervix and urethra.[13] In our population, *C. trachomatis* was detected from both sites in 52.5%, while 35.0% and 12.5% were infected solely in the urethra and cervix, respectively. Over one-third of urogenital chlamydial infections would be missed by failing to sample the urethra; therefore, this site should be evaluated when testing for *C. trachomatis*.

Commonly used laboratory tests to confirm *C. trachomatis* infections include tissue culture and antigen detection techniques. While tissue culture using cycloheximide-treated McCoy cells has long been considered the gold standard diagnostic test for *C. trachomatis*, it will not detect up to 20% of all chlamydial infections.[7] Its clinical use is limited, as it is costly, necessitates stringent transport conditions, including refrigeration, and requires at least 48–72 h of incubation prior to interpretation. Rapid antigen detection tests have been developed using either direct immunofluorescence monoclonal antibody staining or enzyme-linked immunoassay. The sensitivities of these rapid antigen detection tests are less than those of culture. Compared to cell culture, the sensitivities of direct immunofluorescence staining (MicroTrak) and enzyme immunoassay (Chlamydiazyme) range from 61 to 99% and 60 to 98%, respectively.[14] In addition, the low PPVs of the antigen detection tests in low-prevalence populations are concerning. DNA probe assays are additional diagnostic tests available for *C. trachomatis*. The PACE 2 assay (Gen-Probe, Inc., San Diego, CA) is rapid and convenient, but its sensitivity is less than cell culture.[15]

We chose the Amplicor *C. trachomatis* Test, a PCR-based technique, as our gold standard for chlamydia detection, rather than cell culture. The efficacy of PCR in the detection of *C. trachomatis* infections has been documented.[9–12] In a recent study, the Amplicor *C. trachomatis* Test was demonstrated to have a sensitivity of 97% and a specificity of 99.7% in the detection of *C. trachomatis* in cervical specimens, whereas the sensitivity of culture was only 86%.[12] This PCR-based technique is highly sensitive in the detection of cervical chlamydial infections, superior to culture or enzyme-linked immunoassays, and may be a better gold standard for the detection of *C. trachomatis* infections. We chose to compare the Amplicor test to Chlamydiazyme, as Chlamydiazyme is the main test used at our STD clinic and is widely used as the sole diagnostic test for chlamydial infections. Detecting a greater proportion of people infected with *C. trachomatis* has major public health implications. Rapid testing may enable earlier treatment which, theoretically, may prevent the sequelae of chlamydial infections. Moreover, PCR may be shown, in the future, to be a better method to test for cure, differentiating suppression from eradication of the organism. This may be important in adequately treating pelvic inflammatory disease where small inoculum of *C. trachomatis* can persist, causing ongoing tubal damage, perhaps through the immune response to chlamydial heat-shock proteins.[16]

Unlike cell culture, clinical specimens for Amplicor *C. trachomatis* Test analysis can be transported at room temperature, and chlamydial DNA is stable for 6 months when refrigerated in transport medium.[12] The Amplicor system assay is rapid; laboratory processing is only 4.5 h, which compares favorably to the assay times of the antigen detection methods. When chlamydial organisms are present in small amounts, the rapid antigen detection tests are less likely to detect the low levels of antigen present. The PCR method enables the detection of small quantities of specific DNA fragments and has the potential to identify DNA from organisms present in low concentrations. The material and labor costs of this kit depend on the volume of assays performed. In most centers, the total cost will be approximately $20, somewhat more than the cost of Chlamydiazyme but much less than that of cell culture.

Our study demonstrates that the Amplicor *C. trachomatis* Test, a PCR-based process for the detection of *C. trachomatis,* is a rapid and simple diagnostic test for the detection of female urogenital chlamydial infections and is superior to Chlamydiazyme in detecting endocervical infections in an STD clinic population.

### ACKNOWLEDGMENTS

This study was partially supported by Roche Molecular Systems, Branchburg, NJ.

### REFERENCES

1. Sweet RL, Gibbs RS: Infectious Diseases of the Female Genital Tract. 2nd Ed. Baltimore: Williams & Wilkins, p 49, 1990.
2. Sweet RL, Landers DV, Walker C, Schachter J: *Chlamydia trachomatis* infection and pregnancy outcome. Am J Obstet Gynecol 156:824–833, 1987.
3. Wager GP, Martin DH, Koutsky L, et al.: Puerperal infectious morbidity: Relationship to route of delivery and to antepartum *Chlamydia trachomatis* infection. Am J Obstet Gynecol 138:1028–1033, 1980.
4. Gravett MG, Nelson HP, Deroven T, Critchlow C, Eschenbach DA, Holmes KK: Independent associations of bacterial vaginosis and *Chlamydia trachomatis* infection with adverse pregnancy outcome. JAMA 256:1899–1903, 1986.
5. Holmes KK, Handsfield HH, Wang S-P, et al.: Etiology of nongonococcal urethritis. N Engl J Med 292: 1199–1206, 1975.
6. Mardh P-A, Paavonen J, Poulakkainen M: Chlamydia. New York: Plenum Press, p 151, 1989.
7. Mardh P-A, Paavonen J, Poulakkainen M: Chlamydia. New York: Plenum Press, p 79, 1989.
8. Barnes RC: Laboratory diagnosis of human chlamydial infections. Clin Microbiol Rev 2:119–136, 1989.
9. Griffais R, Thibon M: Detection of *Chlamydia trachomatis* by the polymerase chain reaction. Res Microbiol 140: 139–141, 1989.
10. Ostergaard L, Birkelund S, Christiansen G: Use of polymerase chain reaction for detection of *Chlamydia trachomatis.* J Clin Microbiol 28:1254–1260, 1990.
11. Bobo L, Coutlee F, Yolken RH, Quinn T, Viscidi RP: Diagnosis of *Chlamydia trachomatis* cervical infection by detection of amplified DNA with an enzyme immunoassay. J Clin Microbiol 28:1968–1973, 1990.
12. Loeffelholz MJ, Lewinski CA, Silver SR, et al.: Detection of *Chlamydia trachomatis* in endocervical specimens by polymerase chain reaction. J Clin Microbiol 30:2847–2851, 1992.
13. Stamm WE, Holmes KK: *Chlamydia trachomatis* infections of the adult. In Holmes KK, Mardh PA, Sparling PF, et al. (eds): Sexually Transmitted Diseases. 2nd Ed. New York: McGraw-Hill, p 185, 1990.
14. Stamm WE: Diagnosis of *Chlamydia trachomatis* genitourinary infections. Ann Intern Med 108:710–717, 1988.
15. Clarke LM, Sierra MF, Daidone BJ, Lopez N, Covino JM, McCormack WM: Comparison of the Syva Microtrak enzyme immunoassay and Gen-Probe PACE 2 with cell culture for diagnosis of cervical *Chlamydia trachomatis* infection in a high-prevalence female population. J Clin Microbiol 31:968–971, 1993.
16. Morrison RP, Manning S, Caldwell HD: Immunology of *Chlamydia trachomatis* infections: Immunoprotective and Immunopathogenetic Responses. In Quinn TC (ed): Sexually Transmitted Diseases. New York: Raven Press, p 57, 1992.
